# The insulin-like growth factor system is modulated by exercise in breast cancer survivors: a systematic review and meta-analysis

**DOI:** 10.1186/s12885-016-2733-z

**Published:** 2016-08-25

**Authors:** José Francisco Meneses-Echávez, Emilio González Jiménez, Jacqueline Schmidt Río-Valle, Jorge Enrique Correa-Bautista, Mikel Izquierdo, Robinson Ramírez-Vélez

**Affiliations:** 1 Centro de Estudios en Medición de la Actividad Física (CEMA), Escuela de Medicina y Ciencias de la Salud, Universidad del Rosario, Bogotá, DC Colombia; 2 Departamento de Enfermería. Facultad de Ciencias de la Salud, Universidad de Granada, Granada, Spain; 3 Department of Health Sciences Public, University of Navarra, Pamplona, Spain

**Keywords:** Breast cancer, Exercise, Insulin-Like Growth Factor Binding Proteins, Tumor Microenvironment

## Abstract

**Background:**

Insulin-like growth factors (IGF´s) play a crucial role in controlling cancer cell proliferation, differentiation and apoptosis. Exercise has been postulated as an effective intervention in improving cancer-related outcomes and survival, although its effects on IGF´s are not well understood. This meta-analysis aimed to determine the effects of exercise in modulating IGF´s system in breast cancer survivors.

**Methods:**

Databases of PuMed, EMBASE, Cochrane Central Register of Controlled Trials, EMBASE, ClinicalTrials.gov, SPORTDiscus, LILACS and Scopus were systematically searched up to November 2014. Effect estimates were calculated through a random-effects model of meta-analysis according to the DerSimonian and Laird method. Heterogeneity was evaluated with the *I*^2^ test. Risk of bias and methodological quality were evaluated using the PEDro score.

**Results:**

Five randomized controlled trials (*n* = 235) were included. Most women were post-menopausal. High-quality and low risk of bias were found (mean PEDro score = 6.2 ± 1). Exercise resulted in significant improvements on IGF-I, IGF-II, IGFBP-I, IGFBP-3, Insulin and Insulin resistance (*P* < 0.05). Non-significant differences were found for Glucose. Aerobic exercise improved IGF-I, IGFBP-3 and Insulin. No evidence of publication bias was detected by Egger´s test (*p* = 0.12).

**Conclusions:**

Exercise improved IGF´s in breast cancer survivors. These findings provide novel insight regarding the molecular effects of exercise on tumoral microenvironment, apoptosis and survival in breast cancer survivors.

**Electronic supplementary material:**

The online version of this article (doi:10.1186/s12885-016-2733-z) contains supplementary material, which is available to authorized users.

## Background

Insulin-like growth factors (IGFs) are mitogens involved in regulating cell proliferation, differentiation, and apoptosis [1]. The IGF system includes the single-chain polypeptides IGF-I and IGF-II and six binding proteins (IGFBP-I - IGFBP-6) [2]. The IGFBP proteases may also be considered as part of the IGF system because they indirectly regulate the action of IGFs [3]. The IGF family has been linked to several metabolic and disease states, including type 1 diabetes and cancer, especially of the lung, breast, and prostate [3–6]. Both IGF-I and IGF-II exert mitogenic and antiapoptotic actions and regulate tumor cell proliferation and differentiation [3], whilst IGFBP-3 regulates the mitogenic action of IGFs and inhibits their antiapoptotic effects in breast cancer cells due to IGF- inhibitory effects on breast cancer cell growth [7]. In addition, high levels of IGFBP-3 has been associated with low concentrations of estrogen receptor (ER) or progesterone receptor and large tumor size, suggesting a poor prognosis and decreased survival in cancer patients [8, 9].

Exercise has been proposed as an effective non-pharmacological intervention to promote psychological well-being during and following cancer treatment [10–12]. However, the role of exercise in the modulation of the IGF system remains poorly understood and experimental evidence has emerged. At the same time, other researchers have proposed that exercise can be used as a mechanism to decrease IGF levels and aid in cancer prevention [13, 14].

Numerous studies have reported higher levels of circulating IGF associated with physical activity, although many other studies have reported no difference or even a decrease in IGF levels. For example, in 2009, Irwin et al. [15] reported significant reductions in IGF-I and IGFBP-3 in postmenopausal women after a 6-month walking-based intervention compared to non-exercisers. However, Sprod et al. [16] found no significant changes in IGFBP-I and IGFPB-3 after a 12-week intervention of Tai Chi Chuan in twenty-one breast cancer survivors. A limited comprehensive summary has been published that systematically reviews all literature on this topic. In light of this lack of consensus in the literature, the aim of this meta-analysis was to determine the effects of exercise in modulating the IGF system in breast cancer survivors.

## Methods

We followed the Preferred Reporting Items for Systematic Reviews and Meta-Analyses Statement to conduct this review [17]. No funding was received. The PubMed, EMBASE, Cochrane Central Register of Controlled Trials, EMBASE, ClinicalTrials.gov, SPORTDiscus, LILACS and Scopus databases were systematically searched between May and November 2014 by three blinded authors (JFME, JSRV and EGJ) without restrictions on language. The reviewers were blinded to both the name of the authors and the results of the studies. The following search terms were used: ´breast cancer´ and ´exercise´ or ´physical activity´ and ´insulin´ or ´glucose´ or ´growth factors´ or ´IGF´ or ´IGFBP´. The reference lists from retrieved articles were checked to identify additional titles. The authors also examined data from previous reviews published by Ballard-Barbash et al. [18] and Löf and colleagues [19]. Moreover, two authors (MI and RR-V) searched for other relevant trials listed in journals that specialized in oncology (e.g., *BMC Cancer*, *Breast Cancer Research, Cancer*, *Cancer Epidemiology, Biomarkers & Prevention, Journal of Clinical Oncology*, *Journal of Oncology Practice* and *The Lancet Oncology*). Aiming to provide stronger sensitivity to the search process, the authors contacted high-profile researchers in this area to ask for other possibly relevant trials, published or unpublished.

### Selection criteria

Two authors (JSR-V and JEC-B) independently checked all of the retrieved trials against the eligibility criteria (Table 1). The title and abstract were examined, and full-text was obtained if ambiguity regarding the eligibility of the study was noted. A third author arbitrated the consensus for eligibility (EG-J). Attempts were made to contact authors of trial reports if clarification was necessary.

**Table 1:** Inclusion criteria considered in the systematic review

Design • Randomized controlled trial Participants • Women with breast cancer, without restriction to a particular stage of diagnosis or treatment Intervention • Exercise training (i.e., aerobic, resistance training, stretching exercises and Tai Chi Chuan). Outcome measures • Insulin-like growth factor -1 (IGF-1) • Insulin-like growth factor -2 (IGF-2) • Insulin-like growth factor-binding protein -1 (IGFBP-1) • Insulin-like growth factor-binding protein -3 (IGFBP-3) • Insulin • Insulin Resistance • Glucose Comparisons • Exercise training versus conventional care	

A cancer survivor was defined as a person who is diagnosed with cancer and survives from the time of diagnosis through the balance of his or her life [20]. Exercise interventions were defined as a form of physical activity that is planned, structured and repetitive and aims to improve fitness, performance or health [21]. Hence, we included randomized controlled trials (RCTs) that compared exercise interventions (aerobic, resistance training and stretching exercises such as Tai Chi Chuan) with a control group (conventional care) in women with breast cancer and that measured the following biomarkers: insulin-like growth factors (IGF-I and IGF-II), insulin-like growth factor-binding protein (IGFBP-I and IGFBP-3), and insulin serum levels as well as insulin resistance and glucose. This set of biomarkers was selected because they play a vital role in the tumoral microenvironment and cancer prognosis [22, 23]. Finally, we excluded trials where exercise was combined with pharmacological interventions.

### Methodological quality assessment

The methodological quality of the studies including their risk of bias was assessed using the Physiotherapy Evidence Database (PEDro) scale [24]. The PEDro scale scores the methodological quality of randomized trials and has a maximum possible score of 10. Scores were based on all information available from both the published version and from communication with the authors. A score of 5 of 10 was set as the minimum score for inclusion in the review. The score for each included study was determined by two trained authors (JFM-E and MI). Disagreements were solved by consensus or by a third reviewer (JEC-B). We calculated the inter-observer agreement using the *Kappa (k)* statistic [25]; the agreement rate between authors was *k* = 0.91 for methodological quality assessment.

### Data extraction and analysis

Relevant data were extracted independently by two reviewers (JFM-E and RR-V) using a standard form and a third author (JEC-B) mediated in cases of disagreement. The reviewers extracted information about the methods (i.e., design, breast cancer staging, participants and interventions) and the outcome data for the experimental and control groups. High agreement was observed between reviewers (*k* = 0.89).

Changes in the Insulin-Like Growth Factors were reported as differences between arithmetic means pre and post exercise interventions. Statistical heterogeneity was evaluated using the I^*2*^ statistic (*I*^*2*^ = [(*Q* - *df*) / *Q*] X 100 %, where *Q* is the chi-square statistic and *df* is its degrees of freedom), which was defined according to the following categories [26]: negligible heterogeneity, 0 % – 40 %; moderate heterogeneity, 30 % – 60 %; substantial heterogeneity, 50 % – 90 %; and considerable heterogeneity, 75 % – 100 %. Other possible sources of heterogeneity were evaluated via subgroup analysis and a cumulative meta-analysis model if necessary. We conducted a random-effects model of the meta-analysis when substantial heterogeneity (*I*^*2*^ > 50 %) was present. Continuous outcomes were reported as the Standardized Mean Difference (SMD) with the 95 % confidence interval (95 % CI), with statistical significance set at a *P* < 0.05. All analyses were weighted by the inverse variance. Publication bias was examined using Egger´s test (*P* < 0.05) and the funnel plot based on the number of studies included (i.e. if more than 10 trials were included). Based on data availability, we conducted subgroup analysis to explore the particular effects of the modes of exercise separately. All analyses were conducted by JFM-E using Stata (Version 12.0; Stata Corp, College Station, TX).

## Results

### Characteristics of the studies included

**Fig. 1:**
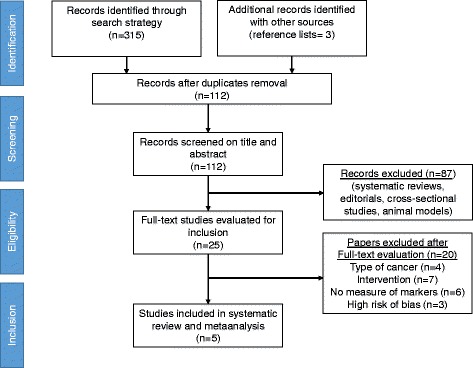
Flowdiagram for search strategy methods. Flowdiagram is performed according to Additional file 1 Statement

A total of five randomized controlled trials (*n* = 235) were included [15, 16, 27–29]. Figure 1 presents the Additional file 1 flow diagram. All groups were similar at baseline with 113 women allocated to the experimental groups and 122 women allocated to the control groups. The average publication date was 2008 ± 3.5 years. An enzyme-linked immunosorbent assay (ELISA) was used by all studies included.

### Methodological quality and risk of bias assessment

We found a high-quality and low risk of bias (mean PEDro score = 6.2 ± 1) across studies. No study performed blinding of participants or therapists and three trials (60 %) blinded their assessors for the analyses (Table 2).

**Table 2:** PEDro Scale scores for the included trials (*n* = 5)

*Study*	*Random allocation*	*Concealed allocation*	*Groups similar at baseline*	*Participant blinding*	*Therapist blinding*	*Assessor Blinding*	*<15 % dropouts*	*Intention to treat analysis*	*Between-group difference reported*	*Point estimate and variability reported*	*Total (0 to 10)*
Fairey et al. [26] (2003)	Y	N	Y	N	N	Y	Y	Y	Y	Y	7
Irwin et al. [14] (2009)	Y	N	Y	N	N	Y	Y	Y	Y	Y	7
Janelsins et al. [27] (2011)	Y	Y	Y	N	N	N	N	N	Y	Y	5
Schmitz et al. [28] (2005)	Y	N	Y	N	N	Y	Y	N	Y	Y	6
Sprod et al. [15] (2012)	Y	Y	Y	N	N	N	N	Y	Y	Y	6
*Compliance rate*	100 %	40 %	100 %	0 %	0 %	60 %	60 %	60 %	100 %	100 %	100 %

*N* No, *Y* Yes, *PEDro* Physiotherapy Evidence Database

### Characteristics of the participants

Most women were postmenopausal, with an average age of 48 ± 3.2 years (range 48-59 years), and were classified with tumor stages I-IIIA after anti-cancer treatment. Chemotherapy was the most common treatment (*n* = 176) followed by radiotherapy (*n* = 124), and 57 participants received hormonal therapy, with the majority of participants receiving tamoxifen. Finally, 133 women received mastectomy and 104 were treated through lumpectomy.

### Characteristics of the exercise interventions

The interventions had a mean length of 22.2 ± 13.5 weeks with an average of 2.8 ± 0.5 sessions per week. The longest exercise intervention length was 12 months reported by Schmitz et al. [29]. The mean session duration was 73 ± 9.6 min. Exercise interventions included aerobic exercise (i.e., walking and stationary cycling) in 2 trials (40 %) [15, 26], resistance training (i.e., strength training) was implemented by Schmitz et al. [29] and Tai Chi Chuan exercises were implemented in two trials [16, 27]. The training intensity varied considerably among studies, ranging from 50 % to 90 % of the maximum heart rate. The adherence rate was 83.7 ± 8.7 %. No major adverse effects were reported. Finally, all studies reported pre-exercise screening before high intensity physical training. Table 3 summarizes the characteristics of the included studies.

**Table 3:** Characteristics of the five randomized controlled trials included in the systematic review and meta-analysis

Study ID	Design	Stage of Disease	Participants	Interventions	Outcome measures
Fairey et al. [26] (2003)	RCT	Breast Cancer Stage I –IIIB	Characteristics of cancer treatment = Women who had completed surgery, radiotherapy, and/or chemotherapy. *N* = 53 Female = 53 Exp (*n* = 25) Age (yr) = 59 (5) Con (*n* = 28) Age (yr) = 58 (6)	Exp = Aerobic exercise. Length = 15 weeks. Duration = Exercise began at 15 min for weeks 1–3, and then systematically increased by 5 min every 3 weeks thereafter to 35 min for weeks 13–15. Frequency = 3 ses/wk. Intensity = 70 %-75 %. Con = Conventional care.	Godin Leisure-Time Exercise Questionnaire, fasting blood.
Irwin et al. [14] (2009)	RCT	Breast Cancer Stage 0-IIIA	Characteristics of cancer treatment = Women who had completed surgery, radiotherapy, and/or chemotherapy. *N* = 68 Female = 68 Exp (*n* = 36) Age (yr) = 56.4 (9.5) Con (*n* = 32) Age (yr) = 55.6 (7.7)	Exp = A combined supervised training program at a local health club and a home aerobic training program. Length = 24 weeks. Duration = 129 min/wk. Frequency = Participants exercised three times per week and were instructed to exercise two days/ week on their own, either at the health club or home. Intensity = Moderate-intensity. Con = Conventional care. Duration = 45 min/wk.	Ainsworth’s Compendium of Physical Activities and fasting blood.
Janelsins et al. [27] (2011)	RCT	Breast Cancer Stage 0-IIIb	Characteristics of cancer treatment = Surgery with axillary lymphadenectomy and both post-surgery radiotherapy and chemotherapy. Female = 19 Exp (*n* = 9) Age (yr) = 54.33 (10.64) Con (*n* = 10) Age (yr) = 52.70 (6.67)	Exp = Tai Chi Chuan (TCC). Length = 12 weeks. Duration = 60 min/session Frequency = 3 session/week. Intensity = Moderate or vigorous. Con = Psychosocial therapy	Bioelectrical impedance tests, Fasting blood.
Schmitz et al. [28] (2005)	RCT	Breast Cancer Stage I-III	Characteristics of cancer treatment = Radiation treatment, chemotherapy, axillary dissection, and hormonal therapy *N* = 85 Female = 85 Exp (*n* = 33) Age (yr) = 53.3 (8.7) Con (*n* = 36) Age (yr) = 52.8 (7.6)	Exp = weight training Length = 12 month (26 weeks) Duration = 60 min each session Frequency = twice-weekly Intensity = moderate Con = conventional care 0-6 month weight training 7-12 month	Body weight, height, body fat, lean mass, body fat %, and waist circumference, as well as fasting glucose, insulin, insulin resistance, insulin-like growth factor-I (IGF-I), IGF-II, and IGF-binding protein-1, IGFBP-2, and IGFBP-3.
Sprod et al. [16] (2012)	RCT	Breast Cancer Stage 0–IIIb	Characteristics of cancer treatment = Surgery (lymphadenectomy and mastectomy) post-surgery radiotherapy and chemotherapy. *N* = 19 Female = 19 Exp (*n* = 9) Age (yr) = 54.33 (3.55) Con (*n* = 10) Age (yr) = 52.70 (2.11)	Exp = Tai chi chuan exercise. Length = 12 weeks. Duration = 60 min/ses. Frequency = 3 ses/wk. Intensity = low to moderate Con = Standard support therapy control (SST)	Cytokine levels and fasting blood.

*RCT* Randomized Controlled Trial, *Exp* Experimental Group, *Con* Control Group

Data are presented as mean (SD)

### Effects of exercise on insulin-like growth factors (IGFs) and their binding proteins (IGFBP-I and IGFBP-3)

**Fig. 2:**
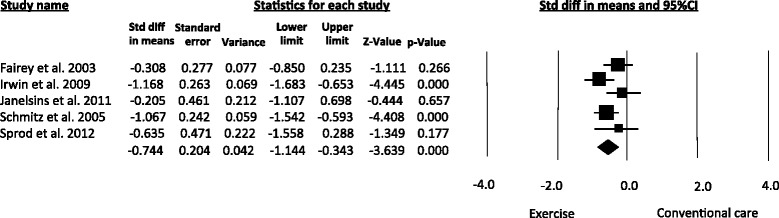
Meta-analysis for the effect estimate of exercise on circulating levels of IGF-I. Standardized Mean Difference (SMD) was calculated for the Random effects model of meta-analysis

Changes in the circulating levels of IGF-I after exercise training were evaluated in five studies [15, 16, 26–29]. The pooled SMD was -0.74 (95 % CI -1.14 to -0.34; *I*^*2*^ = 52.8 %), indicating a moderate reduction in IGF-I following exercise (Fig. 2).

**Fig. 3:**
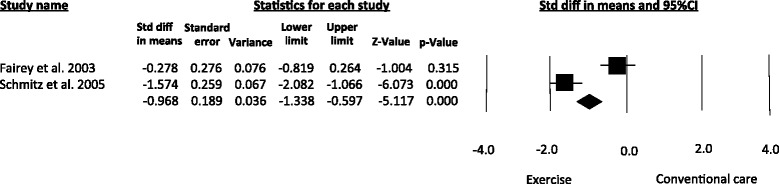
Meta-analysis for the effect estimate of exercise on circulating levels of IGF-II. Standardized Mean Difference (SMD) was calculated for the Random effects model of meta-analysis

Similar improvements were obtained for IGF-II (SMD = -0.96, 95 % CI -1.33 to -0.59; *I*^*2*^ = 91.4 %) [26, 28] (Fig. 3), which was measured in two studies [26, 28]. These estimates were obtained using a random-effects model. A meta-regression analysis to explore dose–response relationships was not conducted due to the limited number of studies included.

**Fig. 4:**
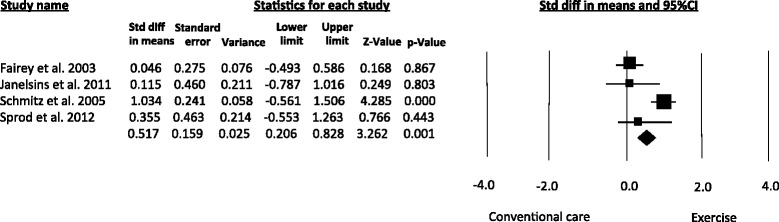
Meta-analysis for the effect estimate of exercise on circulating levels of IGFBP-I. Standardized Mean Difference (SMD) was calculated for the Random effects model of meta-analysis

Based on data from four articles [15, 26–28], the pooled estimates revealed that exercise improved the serum levels of Insulin-like growth factor-binding protein-I (IGFBP-I) (SMD = 0.51, 95 % CI 0.20 to 0.82; *I*^*2*^ = 62 %) (Fig. 4).

**Fig. 5:**
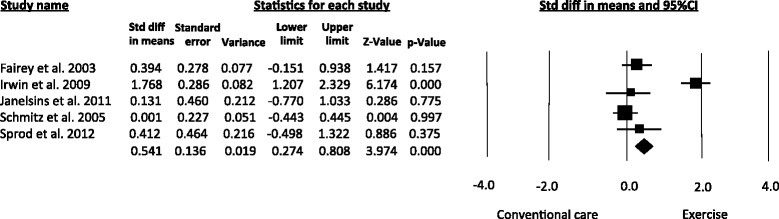
Meta-analysis for the effect estimate of exercise on circulating levels of IGFBP-3. Standardized Mean Difference (SMD) was calculated for the Random effects model of meta-analysis

All of the studies included [15, 16, 26–28] evaluated the serum concentrations of IGFBP-3 and demonstrated that exercise training significantly increased the serum levels of this biomarker in women with breast cancer (SMD = 0.54, 95 % CI 0.27 – 0.80; *I*^*2*^ = 84.2 %) (Fig. 5).

**Fig. 6:**
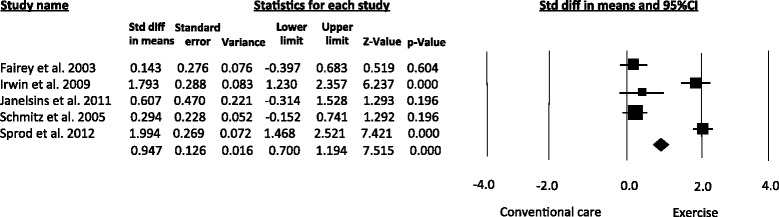
Meta-analysis for the effect estimate of exercise on circulating levels of insulin. Standardized Mean Difference (SMD) was calculated for the Random effects model of meta-analysis

In addition, exercise interventions resulted in significant differences in the levels of insulin (SMD = 0.94, 95 % CI 0.70 – 1.19; *I*^*2*^ = 93.8 %) (Fig. 6) and insulin resistance (SMD = -0.35, 95 % CI -0.70 to -0.009; *I*^*2*^ = 0 %). Non-significant differences were obtained for glucose levels (SMD = -0.16, 95 % CI -0.43 to 0.10; *I*^*2*^ = 0 %).

### Subgroup analysis by mode of exercise

**Fig. 7:**
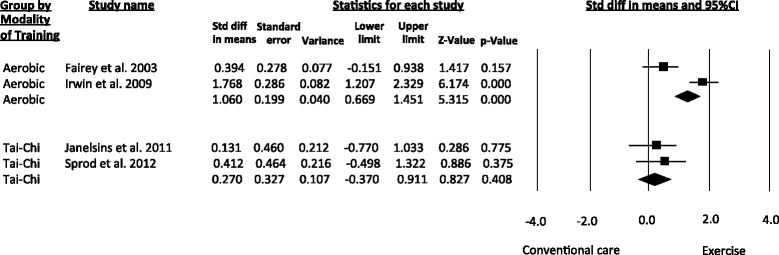
Meta-analysis for subgroup analysis by mode of exercise. Standardized Mean Difference (SMD) was calculated for the Random effects model of meta-analysis

Regarding the subgroup analyses, aerobic exercise improved the serum concentrations of IGF-I, IGFBP-3 and insulin. Aerobic exercise analysis for IGFBP-I was not possible because only Fairey et al. [26] evaluated this marker. Tai-Chi training resulted in significant benefits for insulin levels. Tai-Chi also improved the serum concentrations of IGF-I, IGFBP-I and IGFBP-3, although these effects did not reach significance. Resistance training analysis was not conducted because only Schmitz et al. [29] evaluated this mode of exercise. Subgroup analysis for IGF-II was not possible because the two studies that measured this marker implemented different exercise modes [26, 28]. Figure 7 displays the subgroup analysis according to mode of exercise for IGFBP-3. Table 4 describes the effect estimates for the subgroup analyses undertaken in the meta-analysis.

### Publication bias

No evidence of publication bias was detected by Egger´s test (*P* = 0.12); a funnel plot was not built due to the limited number of studies included in the pooled analysis.

## Discussion

The most remarkable finding from this meta-analysis was that exercise training improved the serum levels of IGF-I, IGF-II, IGFBP-I and IGFBP-3 in breast cancer survivors after successful anticancer treatment. Similar conclusions have been reported in previous experimental studies [15, 26, 28]. Moreover, it is important to highlight that this is the first meta-analysis that has summarized the effectiveness of exercise training in modulating the IGF system in breast cancer survivors because a previous systematic review regarding exercise and blood biomarkers in breast cancer survivors was published by Löf and colleagues [19], but the authors did not undertake data synthesis analysis.

The mitogenic and antiapoptotic effects of IGF-1 are related to a poorer prognosis in breast cancer [30] and increased all-cause mortality [31]. The type of exercise did not appear to affect any putative association; however, it is probable that different exercise modalities cause different responses in IGF-1. Our pooled analysis demonstrated that exercise reduced IGF-I concentrations in women with breast cancer after successful treatment. These findings are consistent with those by some studies included in our meta-analysis, such as the trial published by Fairey et al. [26], in which a 15-week aerobic exercise intervention resulted in significant decreases in IGF-I levels (10.9 %) in fifty-three postmenopausal breast cancer survivors. Data from a Yale study [15] also confirm our findings; in this study, the authors found an 8.9 % significant reduction in IGF -I in an experimental group composed of 38 breast cancer survivors that completed 150 min/wk of moderate intensity aerobic exercise during 5 weeks compared to a control intervention (i.e., instructions for patients to maintain their current physical activity level). Another consideration in assessing studies using an exercise intervention is the timing of blood sampling in relation to exercise. Most studies that have demonstrated a post-exercise increase in IGF-1 found an immediate post-exercise spike followed by a gradual return to baseline or lower than baseline IGF -1 levels over the next 30 min to several hours [13].

It has been demonstrated that IGFBP-3 restricts IGF-1 availability and biological activity [32] and thus, low levels of IGFBP-3 have been associated with an increased risk of breast cancer [33] and a poorer prognosis and have been postulated as predictors of distant recurrence of breast carcinoma in postmenopausal women [1, 34]. We found that exercise training increased IGFBP-3 serum levels in breast cancer survivors, although high statistical heterogeneity was observed in the overall effect estimate (*I*^*2*^ = 84.2 %). We obtained similar results for the aerobic exercise subgroup analysis. These findings are consistent with those published by Fairey et al. [26] and Irwin et al. [15] from the Yale study described above. In addition, when adjusted by exercise mode in the subgroup analysis, we found that Tai Chi was an effective intervention in increasing IGFBP-3 serum levels in breast cancer survivors, although statistical significance was not reached. Similar results were published by Janelsins et al. [28] in a randomized controlled trial in 19 breast cancer survivors, where a 12-week exercise intervention of Tai Chi increased IGFBP-3 serum levels compared to non -exercise. Conversely, non-significant changes in IGFBP-3 were observed by Sprod et al. [16] in a more recent study with a similar intervention using Tai Chi. Interestingly, the authors reported an association between changes in IGFBP-3 and physical functioning, suggesting a link between changes in IGF binding proteins and some domains of quality of life in breast cancer survivors, although these associations warrant additional research. However, several studies that have reported a change in IGFBP-3 following an acute exercise challenge usually found a pattern similar to that found for total IGF-1 [13].

Regarding the secondary outcomes of this meta-analysis, our analyses showed that exercise produces significant increases in insulin and significant decreases in the insulin resistance of breast cancer survivors; reductions in the glucose levels did not reach statistical significance. Subgroup analysis by mode of exercise was limited for insulin and insulin resistance due to the number of studies included. Similar to our results, Sprod et al. [16] reported slight increases in insulin levels after a Tai Chi intervention. Nonetheless, other studies have reported mixed findings. Schmitz et al. [29] found no changes in insulin or glucose after weight training exercise in 85 breast cancer survivors; Ligibel et al. [35] detected significant reductions in insulin levels after a twice-weekly resistance training intervention for 16 weeks in breast cancer survivors. Lastly, Irwin et al. [15] stated that the lack of changes in insulin and glucose levels can be affected by weight status at baseline (i.e., obese breast cancer survivors have higher insulin levels than participants with normal or lower weight), suggesting that heavier participants can benefit more from exercise compared to leaner participants with respect to changes in glycemic control.

In this sense, several biologically plausible mechanisms could explain the effects of exercise in modulating the IGF and IGFBP systems. It is widely known that exercise has the potential to reduce both hepatic and muscle insulin resistance and to increase glucose availability due to insulin signaling pathways, improvements in capillary density leading to a better delivery of muscle glucose, increases in glucose protein transporters and effects on mRNA [36]. These conditions decrease the insulin concentration due to lower concentrations of IGFs via insulin-mediated changes in IGFBP concentrations [14]. However, further research is needed to confirm these mechanisms, especially in breast cancer survivors during and after anticancer treatment regimens, and gain insight regarding the benefits that exercise and multidimensional behavioral change interventions can provide on cancer treatment related outcomes and survival, moving from preventive strategies toward patients facing cancer.

Only one study examined the effects of resistance training alone, and this method was also beneficial [29]. The effects of resistance exercise have not been addressed by the American Cancer Society but have been examined recently in people undergoing cancer treatment [37]. However, the present meta-analysis indicates that further evidence regarding the effects of resistance training during and after anticancer treatment. Besides, to understand the possible mechanisms, more information is required regarding the effects of initial chemotherapy and radiation therapy on muscle satellite (progenitor) cells that proliferate in response to resistance exercise [10, 11].

### Strengths and limitations

To our knowledge, this is the first meta-analysis that evaluates the changes on insulin-like growth factors and their binding proteins after exercise training in breast cancer survivors. Our results provide novel insight regarding the role of exercise as a non-pharmacological and non-cytotoxic effective intervention in modulating the tumoral microenvironment as well as in the management of cancer treatment-related side effects (i.e., fatigue, depression and impairments of quality of life). In addition, there were numerous methodological limitations that impacted the generalizability of studies, including a lack of adjustment for confounding factors (e.g., plasma volume, participant age or body composition) and a lack of consideration of effect modification [13]. Furthermore, our findings have crucial implications on cancer recurrence and disease free survival rates. In addition, all studies included exhibited moderate to high methodological quality and low risk of bias, which is an important issue in terms of external validity.

Nevertheless, some limitations with regard to our study exist that are important to state. The overall effects estimates were increased due to different modes of exercise across the studies included, although such differences were approached through subgroup analysis according to the mode of exercise. High statistical heterogeneity levels were detected for most of the effect estimates, which suggests some caution when interpreting our findings. This evidence of heterogeneity was counteracted by a random effects model of analysis and can be explained by differences in some characteristics of exercise such as intensity, duration, intervention length, follow up periods and adherence rates across studies. Furthermore, dose–response relationships were not explored due to the number of studies included, and further trials might provide specific details regarding training intensity, duration and length of exercise interventions in order to strength the consensus in this field. Finally, considering that all studies involved women who completed their therapeutic treatments, it is important that further studies include patients during the active treatment stages to elucidate the effects of exercise on IGFs in patients undergoing anti-cancer treatment.

## Conclusions

Exercise training is an effective and safe intervention for the improvement of serum levels of the IGF system and its binding proteins (IGFBP-I and IGFBP3) as well as for insulin and glucose control in breast cancer survivors, suggesting a beneficial role of exercise for the tumoral microenvironment and breast cancer recurrence and disease free survival rates in women with breast malignancies. Important components for future research have been identified that should address many of the limitations found in the reviewed studies, which would advance this area of research by answering questions on exercise, IGFs, and health, an area that is growing in interest and importance. High-quality studies are necessary to determine an optimal exercise program and to assess the clinical relevance of the results of available research.

## Additional file:

Additional file 1: PRISMA 2009 Checklist. (DOCX 34 kb)

## Abbreviations

CI: Confidence interval
ELISA: Enzyme-linked immunosorbent assay
IGF: Insulin-like growth factors
IGFBP: Insulin-like growth factor binding protein
MD: Mean difference
PEDro: Physiotherapy evidence database
RCT: Randomized controlled trials
SD: Standard deviation
